# Geminivirus-Encoded Proteins: Not All Positional Homologs Are Made Equal

**DOI:** 10.3389/fmicb.2020.00878

**Published:** 2020-05-05

**Authors:** Ana P. Luna, Rosa Lozano-Durán

**Affiliations:** ^1^Instituto de Hortofruticultura Subtropical y Mediterránea “La Mayora” (IHSM-UMA-CSIC), Area de Genética, Facultad de Ciencias, Universidad de Málaga, Málaga, Spain; ^2^Shanghai Center for Plant Stress Biology, CAS Center for Excellence in Molecular Plant Sciences, Chinese Academy of Sciences, Shanghai, China

**Keywords:** geminivirus, viral protein, positional homolog, silencing suppressor, C2/AC2, V2/AV2, C4/AC4

## The Plant Geminiviruses

**Figure 1:**
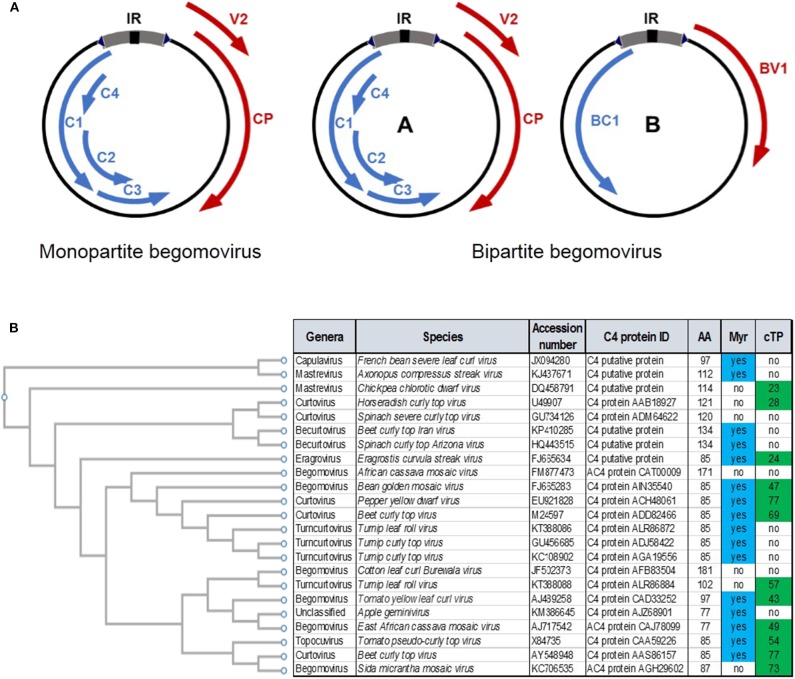
**(A)** Geminivirus (begomovirus) genome structure in monopartite and bipartite species. Arrows represent open reading frames (ORFs). ORFs in the virion strand are in red; ORFs in the complementary strand are in blue. See text for details. **(B)** Comparison of the C4 proteins from different geminivirus species across genera. The presence of a predicted myristoylation site (Myr) or chloroplast transit peptide (cTP) in the protein sequence is indicated.

Geminiviruses are insect-transmitted plant viruses with circular, single-stranded (ss)DNA genomes that cause devastating diseases in major crops worldwide. The family *Geminiviridae* comprises more than 450 species divided in nine genera, based on genome organization, host range, and insect vector: *Begomovirus, Mastrevirus, Curtovirus, Becurtovirus, Topocuvirus, Turncurtovirus, Capulavirus, Gablovirus*, and *Eragrovirus* (Zerbini et al., 2017). The most diverse genus in this family is *Begomovirus*, which to date includes 409 different species (reviewed in Zhao et al., 2019). Begomoviruses can be further subdivided in monopartite, with one-molecule genomes, and bipartite, with two-molecule genomes (Figure 1A). Regardless of whether they are mono- or bi-partite, the size of each genomic DNA molecule is ~3 kb.

Apart from the obvious economic and practical interest propelling the study of geminiviruses, this virus family is an excellent model system to gain insight into plant processes. Geminiviruses replicate their DNA genomes in the nucleus by using the plant DNA replication machinery; the geminivirus genome forms minichromosomes that are subjected to epigenetic modifications; geminiviruses are both activators and suppressors of plant defense responses, and modulate plant developmental processes (reviewed in Hanley-Bowdoin et al., 2013). Therefore, geminiviruses can be used as probes to deepen our understanding not only of plant-virus interactions, but also of different aspects of plant biology.

## Geminivirus-Encoded Proteins

As intracellular parasites, geminiviruses have to effectively manipulate plant cell functions to replicate, suppress anti-viral defense, and move throughout the plant, ultimately establishing a systemic infection; their evolved capacity to co-opt and modulate processes in a given host plant will determine the outcome of the plant-virus interaction. In order to hijack the host cell molecular machinery, geminiviruses produce a limited number (between 4 and 8) of small, fast-evolving, multifunctional proteins, encoded by bidirectional and partially overlapping open reading frames (ORFs) (Figure 1A). Monopartite begomoviruses encode six proteins, namely C1/Rep, C2/TrAP, C3/REn, C4, V2, and V1/CP. Homologs are encoded in one of the genomic component of bipartite begomoviruses, DNA A (in this case, named AC1/Rep, AC2/TrAP, AC3/REn, AC4, AV2, and AV1/CP); the other component in bipartite species, termed DNA B, encodes two additional proteins: the nuclear shuttle protein (NSP) and the movement protein (MP) (Figure 1A). Curiously, monopartite begomoviruses are often found in nature associated with satellite molecules, known as α- and β-satellites, which contribute to or even enable viral pathogenicity through the action of their encoded proteins (α-Rep and β-C1, respectively) (reviewed in Zhou, 2013).

In view of the fast pace of evolution of geminivirus genomes (reviewed in Zhao et al., 2019), it is expected that all proteins therein encoded are essential for the viral infection—since otherwise their coding sequence would be eventually lost. This idea is supported by the results obtained in the laboratory with artificially mutated viruses, which generally present a dramatically decreased virulence in their natural hosts and a high rate of reversion. Our current knowledge of the specific molecular function of individual geminivirus-encoded proteins derives from an ever-growing body of work, carried out by multiple research groups worldwide during the past few decades and resulting from the combination of molecular biology, cell biology, virology, and biochemistry.

Considering the biological properties and life cycle of geminiviruses and plant viruses in general, a series of functions that are *conditio sine qua non* for a successful viral infection can be inferred: these include manipulation of the cell cycle, DNA replication, intra- and inter-cellular movement, and suppression of gene silencing and other anti-viral defenses, such as the response to defense-related hormones. Virus-encoded proteins exerting these functions have indeed been identified in different geminivirus species, although in some cases the exact underlying molecular mechanisms remain to be unraveled (reviewed in Hanley-Bowdoin et al., 2013; Yang et al., 2016).

## Positional Homologs in Geminiviruses

Genome structure is conserved among geminiviral species within the same genus, and in some cases even among species in different genera: genes in the same strand (virion or complementary) and position in different geminivirus species are therefore referred to as positional homologs, have the same name, and the resulting proteins show sequence similarity at the amino acid level (Figure 1). Given these shared properties, together with the observation that the biological requirements for a successful geminivirus infection are most likely common to all family members, positional homologs are frequently considered equivalent, and the properties identified for an individual gene are often extrapolated to others. This notion assumes that positional homologs are invariably and necessarily functional homologs; nonetheless, this is at odds with the idea of functional diversification that could result from the fast adaptation of different virus species to their hosts. Without the intention to be exhaustive, some specific examples are briefly discussed below.

Some functions of positional homologs seem indeed to be conserved across geminivirus species and genera: this is the case of Rep, which facilitates replication of the viral genome in all known species by reprogramming the cell cycle and mediating initiation, elongation, and termination of viral DNA replication (reviewed in Hanley-Bowdoin et al., 2013; Ruhel and Chakraborty, 2019); or that of V2, which acts as a suppressor of post-transcriptional gene silencing (PTGS) in all geminivirus species tested to date (Zrachya et al., 2007; Sharma and Ikegami, 2010; Amin et al., 2011; Zhang et al., 2012; Luna et al., 2017; Yang et al., 2018; Zhan et al., 2018; Mubin et al., 2019). Nevertheless, it has to be considered that geminivirus-encoded proteins are multifunctional: Rep, for example, promotes viral transcription (Kushwaha et al., 2017) and works as a suppressor of either transcriptional gene silencing (TGS) or PTGS in certain species (Rodríguez-Negrete et al., 2013; Liu et al., 2014); some V2 proteins act as suppressors of TGS (Wang et al., 2014, 2018, 2020; Mubin et al., 2019), and inhibit a host protease (Bar-Ziv et al., 2015). Therefore, at this point, whether functional homology among Rep or V2 proteins is complete or only partial is unclear.

On the other hand, examples of geminiviral positional homologs with proven partial functional homology are available in the literature. Perhaps the most illustrative case to date is that of the C2/AC2 proteins: in begomoviruses and curtoviruses, C2/AC2 proteins have a conserved zinc-finger motif, despite showing only limited similarity in the overall amino acid sequence; but while AC2, but perhaps not C2, from begomoviruses acts as a transcriptional activator for viral and some plant host genes (Sunter and Bisaro, 1992, 1997; Wartig et al., 1997; Trinks et al., 2005), C2 from curtoviruses lacks an obvious transcriptional activation domain and transcriptional activation activity (Sunter et al., 1994; Baliji et al., 2007). At least in two species, C2/AC2 interacts with and inactivates SNF1-related kinase (also known as Arabidopsis protein kinase 11 [AKIN11]), a global regulator of metabolism (Hao et al., 2003; Wang et al., 2003). Some C2/AC2 proteins are suppressors of PTGS (Voinnet et al., 1999; Vanitharani et al., 2004; Wang et al., 2005; Luna et al., 2012), but not others (Vanitharani et al., 2004; Luna et al., 2012). C2/AC2 has also been shown to suppress TGS by interfering with the methyl cycle in several species, but through at least two different mechanisms, namely the inhibition of adenosine kinase (ADK) (Buchmann et al., 2009; Jackel et al., 2015) and the attenuation of the proteasome-mediated degradation of S-adenosyl-methionine decarboxylase 1 (SAMDC1) (Zhang et al., 2011). A third strategy to suppress TGS is exhibited by the C2/AC2 protein encoded by at least two other species, of which the C2/AC2 proteins interact with and inhibit the H3K9 histone methyltransferase SUVH4/KYP (Castillo-González et al., 2015; Sun et al., 2015). The C2 protein encoded by a curtovirus creates a cellular environment permissive to DNA replication, but this function is not shared by the protein encoded by the position homologue in begomoviruses (Caracuel et al., 2012; Lozano-Duran et al., 2012) (Table 1).

**Table 1:** Different C2/AC2 functions described in several geminiviral species.

**Virus**	**Function**	**References**
*Tomato golden mosaic virus* (TGMV); *Mungbean yellow mosaic virus* (MYMV)	Transcriptional activator for viral and some plant host genes	Sunter and Bisaro, 1992, 1997; Trinks et al., 2005
*Tomato golden mosaic virus* (TGMV) and *Beet curly top virus* (BCTV)	Inactivation of SNF1-related kinase (*Arabidopsis* protein kinase 11 [AKIN11])	Hao et al., 2003; Wang et al., 2003
*African cassava mosaic virus* (ACMV); *Tomato yellow leaf curl virus* (TYLCV); *Tomato golden mosaic virus* (TGMV); *Beet curly top virus* (BCTV); *Indian cassava mosaic virus* (ICMV) and *East African cassava mosaic Cameroon virus* (EACMCV)	Posttranscriptional gene silencing (PTGS) suppression	Voinnet et al., 1999; Vanitharani et al., 2004; Wang et al., 2005; Luna et al., 2012
*Tomato golden mosaic virus* (TGMV); *Cabbage leaf curl virus* (CaLCuV), and *Beet curly top virus* (BCTV)	Transcriptional gene silencing (TGS) suppression by interfering with the methyl cycle through inhibition of adenosine kinase (ADK)	Buchmann et al., 2009; Jackel et al., 2015
*Beet severe curly top virus* (BSCTV)	TGS suppression by interfering with the methyl cycle through attenuation of the proteasome-mediated degradation of S-adenosyl-methionine decarboxylase 1 (SAMDC1)	Zhang et al., 2011
*Tomato golden mosaic virus* (TGMV); *Cabbage leaf curl virus* (CaLCuV) and *Indian cassava mosaic virus* (strains: ICMV-Dha and ICMV-SG)	TGS suppression by inhibiting the H3K9 histone methyltransferase SUVH4/KYP	Castillo-González et al., 2015; Sun et al., 2015
*Beet curly top virus* (BCTV)	Creation of a cellular environment permissive to DNA replication	Caracuel et al., 2012; Lozano-Duran et al., 2012

The functions of the geminivirus-encoded C4/AC4 could be at least as varied in different species as those of C2/AC2. Several independent functions have been ascribed to C4/AC4 to date (e.g. Piroux et al., 2007; Teng et al., 2010; Luna et al., 2012; Sunitha et al., 2013; Ismayil et al., 2018; Li et al., 2018; Mei et al., 2018, 2020; Rosas-Diaz et al., 2018), and transgenic *Arabidopsis thaliana* plants expressing C4/AC4 from different geminiviruses display distinct developmental phenotypes (Mills-Lujan and Deom, 2010; Luna et al., 2012). Perhaps even more importantly, the C4/AC4 proteins encoded by different geminivirus species can have non-perfectly overlapping subcellular localizations, depending on specific targeting signals, namely acylation sites and a chloroplast transit peptide (e.g., Fondong et al., 2007; Carluccio et al., 2018; Mei et al., 2018; Rosas-Diaz et al., 2018; Zhan et al., 2018; Medina-Puche et al., 2019) (Figure 1B). These differences in subcellular distribution of different C4/AC4 proteins, which can be found associated to membranes, in the cytoplasm, in the nucleus, or in chloroplasts, will in all likelihood have a strong impact on their functionality during infection. Interestingly, C4 is seemingly under positive selection, in stark contrast to other geminiviral proteins (Sanz et al., 1999; Melgarejo et al., 2013; Yang et al., 2014).

In summary, a growing body of experimental data supports the idea that, although positional homologs have a common origin and frequently share functions, this functional overlap is not necessarily complete, since novel roles will have most likely been acquired during evolution. At the same time, not all geminiviral ORFs have positional counterparts (e.g., those in the DNA-B of bipartite geminiviruses), and therefore the essential virulence functions provided by the proteins they encode must be fulfilled by other, non-homologous geminiviral proteins. Hence, caution must be taken when extrapolating functional information to positional homologs, and uncovering the roles of each geminivirus-encoded protein in individual species will in all cases require experimental assessment.

## Author Contributions

AL and RL-D conceived the idea and prepared the manuscript.

## Conflict of Interest

The authors declare that the research was conducted in the absence of any commercial or financial relationships that could be construed as a potential conflict of interest.
